# Diversity of the Alongshan Virus in *Ixodes* Ticks Collected in the Russian Federation in 2023

**DOI:** 10.3390/microorganisms13112564

**Published:** 2025-11-10

**Authors:** Mikhail Y. Kartashov, Kirill A. Svirin, Maria E. Antonets, Alina S. Zheleznova, Valentina Y. Kurushina, Alexander P. Agafonov, Vladimir A. Ternovoi, Valery B. Loktev

**Affiliations:** 1 State Research Center of Virology and Biotechnology “Vector”, Rospotrebnadzor, 630559 Koltsovo, Russia; kartashov_myu@vector.nsc.ru (M.Y.K.);; 2 Specialized Educational Scientific Center, Novosibirsk National Research State University, 1 Pirogov St., 630090 Novosibirsk, Russia; 3 Institute of Cytology and Genetics, Siberian Branch of the Russian Academy of Sciences, 10 Academician Lavrentyev Ave., 630090 Novosibirsk, Russia

**Keywords:** Alongshan virus, flavi-like virus, tick-borne infections, Russia

## Abstract

A novel flavi-like virus with a segmented genome—Alongshan virus (ALSV)—has been isolated from Ixodes ticks in Russia. In this study, 4458 ixodid ticks collected in 22 regions of Russia were tested for genetic markers of ALSV by RT PCR. The highest rates of ALSV infection in ticks were detected in the Republic of Khakassia (3.3%) and in Kemerovo Oblast (2.4%), while low infection rates were more typical in the European part of Russia (0.4–0.7%). Complete four-segment genomes of 20 ALSV isolates derived from 22 PCR-positive *Ixodes persulcatus* ticks were sequenced using a high-throughput approach. The nucleotide sequences for Asian ALSV isolates have a 94.5–96.5% identity to ALSV isolates previously found in China, with this range for the European isolates being 89–93%. This data, together with phylogenetic analysis, indicates the existence of Asian and European subtypes of ALSV, and these may be associated with *I. persulcatus* and *I ricinus* ticks. The obtained results express the spread of ALSV in Russia and also may be useful for the diagnosis, prophylactics, and treatment of this infection.

## 1. Introduction

Tick-borne flaviviruses are widespread throughout the world and pose a serious medical problem, causing a significant number of infectious diseases among people [1]. In Russia, the main tick-borne orthoflaviviruses reported are Powassan virus, Omsk hemorrhagic fever virus, and tick-borne encephalitis virus [2,3,4]. Despite the fairly large species diversity, the genome of all flaviviruses has a typical structure and is a non-segmented ss(+)RNA approximately 11 kb long, encoding one extended open reading frame, at the edges of which are the 5′ and 3′ untranslated regions [5,6].

However, several novel flavi-like viruses have been isolated in recent decades. These viruses are characterized by a segmented genome, a feature that distinguishes them from classical orthoflaviviruses, and they are classified within a distinct taxonomic group, the Jingmenviruses [7,8,9,10]. Such viruses have a segmented positive single-stranded RNA genome, and only two genes have a certain identity to the RNA-dependent RNA polymerase (NS5) and helicase (NS3) of “classical” orthoflaviviruses. This Jingmenvirus group includes the Alongshan virus (ALSV), Jingmen tick, Yanggou tick, Mogiana tick, and Kindia tick viruses, and a number of other viruses [10,11]. To date, these flavi-like viruses have been detected across Asia, Europe, South America, and Africa.

The genomes of segmented flavi-like viruses include either four segments (typical of viruses isolated from ticks, bats, monkeys, and humans) or five segments for viruses isolated from mosquitoes [11]. Segment 1 encodes the NS5-like nonstructural protein, which is similar to NS5 in orthoflaviviruses, and Segment 3 encodes the NS3 polypeptide. The N-terminal domain of NS3 exhibits protease activity, and the C-terminal domain functions as a helicase. The NS3 protein, along with NS5, plays a central role in virus replication. Proteinase activity is required for polyprotein processing, while the helicase domain is involved in capping and viral RNA synthesis. To date, the structure of the NS3 protein in most unsegmented flaviviruses has been studied, and high homology has been shown not only in terms of structure but also in the mechanisms of ATP hydrolysis, recognition, and the unwinding of RNA. Structural proteins VP1, VP2, and VP3 are encoded in Segments 2 and 4 and have no known homologues either among the *Flaviviridae* family or among other known viruses. Segment 2 in ALSV encodes putative glycoproteins VP1a and VP1b, as well as a small protein with three transmembrane domains, the function of which is unknown. Proteins VP2 (putative capsid protein) and VP3 (putative viral membrane protein) are encoded in Segment 4 and have partially overlapping translation frames.

In addition, additional genomic segments have recently been described for the Jingmenvirus genome [12]. This discovery reveals the fluidity of this genome and the possibility of combinations of segments packaged in different virus particles. This may provide additional evidence indicating that multipartite virions really do exist.

Following the discovery of the first known flavi-like viruses with segmented (multipartite) genomes in China and Brazil [7,8], the circulation of ALSV was detected in ticks and humans in northeastern China (Inner Mongolia and Heilongjiang Province) [13,14]. Subsequent studies detected ALSV RNA in *I. ricinus* ticks in Finland, France, Serbia, Germany, and Switzerland [11,15,16,17,18]. ALSV has also been detected in Russia [19,20,21,22]. The genetic material of ALSV has been found in *I. persulcatus*, *I. ricinus*, *Dermacentor reticulatus*, and *D. nuttalli* ticks collected in the Kaliningrad, Ulyanovsk, and Chelyabinsk oblasts, as well as in the Russian Republics of Karelia, Tatarstan, Gorny Altai, and Tuva. The pathogenicity of multicomponent flavi-like viruses for domestic animals and humans has now been proven. However, this information is fragmentary and limited. It is possible that ALSV’s role in infectious pathology may be more significant than is commonly believed.

The aim of this study is to find novel ALSV isolates from ixodid ticks in different regions of Russia and perform whole-genome molecular genetic characterization on them.

## 2. Materials and Methods

### 2.1. Collection and Processing of Ticks

**Figure 1:**
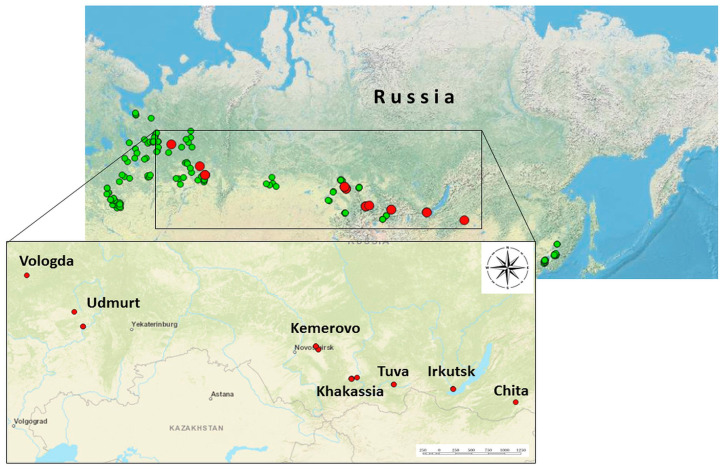
Map of tick collection sites. The sites where ALSV-positive ticks were found are marked by red circles and ALSV-negative ticks—green circles. The precise geographic coordinates for ALSV-positive sites and their descriptions are presented in Tables S1 and S2.

In this study, 4458 individual samples of adult ticks of the species *I. persulcatus* (N = 4122) and *I. ricinus* (N = 336) were analyzed. The ticks were collected in 23 regions in the summer of 2023 by flagging from vegetation. Figure 1 shows the locations of tick collection sites and tick species. The species of tick samples was established by their morphological characteristics. The collected ticks were washed twice with 70% ethanol to remove external contaminants and external microflora, following which they were stored at a temperature of –80 °C for subsequent analysis. Additional taxonomic verification of ALSV-positive tick samples was carried out by determining the nucleotide sequence of the mitochondrial cytochrome oxidase gene.

### 2.2. Reverse Transcriptase PCR (RT-PCR) and Sequencing of Amplified Products

Adult ticks were homogenized using the TissueLyser II laboratory homogenizer (QIAGEN, Hilden, Germany) in 300 µL 0.9% saline solution. Viral RNA from 150 µL tick suspensions was isolated with ExtractRNA (Evrogen, Moscow, Russia), according to the manufacturer’s protocols. Screening of the obtained samples for the presence of ALSV RNA was performed by RT-PCR using screening primers complementary to a fragment of Segment 2: Miass_gly_3F TGGATCAGCTCACACCACAC and Miass_gly_3R TCACCGTCACAGTGGAATGG [19]. PCR was performed on a T1000 amplifier (Bio–Rad, Hercules, CA, USA) in 25 μL of the BioMaster RT-PCR Standard reaction mixture (2×) (Biolabmix, Novosibirsk, Russia) containing 0.4 pM primers under the following conditions: polymerase activation at 95 °C for 5 min and then 38 cycles of 95 °C for 10 s, 53 °C for 20 s, and 68 °C for 30 s. The amplification products (expected length 333 bp) were analyzed by electrophoresis in 2% agarose gel containing ethidium bromide at a concentration of 2 μg/mL and visualized in the UV spectrum using a GelDoc Go Gel Imaging System (Bio–Rad, Hercules, CA, USA). To confirm the specificity of RNA detection, the ALSV PCR product was gel-purified and then sequenced in both directions on the ABI PRISM 3500 (Applied Biosystems, Foster City, CA, USA) sequencer using ABI PRISM^®^ BigDye™ Terminator v.3.1.

### 2.3. NGS Sequencing and NGS Data Analysis

To enrich the library for high-throughput sequencing, we used targeted PCR with a panel of primers for all 4 segments (Table 1). To perform targeted amplification of ALSV, the amplification method was optimized by experimentally choosing the temperature regime and concentrations of the reaction mixture components. The concentration of purified PCR products was estimated by the fluorescence method on a Qubit 2.0 device (Thermo Fisher Scientific, Waltham, MA, USA) using the Qubit dsDNA HS Assay Kit (Thermo Fisher Scientific). Sequencing was performed using the MiSeq Reagent Kit v3 (Illumina, San Diego, CA, USA) for 600 cycles. Cutadapt (version 5.1) and SAMtools (version 1.20) were used to remove the Illumina adaptors and duplicate reads. The contigs were assembled de novo using the MIRA assembler with default parameters (version 4.9.6). High-throughput sequencing data were processed using a BLASTN-based (version 2.17.0+) taxonomic read identification algorithm.

### 2.4. Phylogenetic Analysis

The nucleotide sequences of the genome coding regions of each segment were aligned using ClustalW. Phylogenetic analysis was conducted using the maximum likelihood method and the Tamura–Nei model in MEGA 10/11 with 1000 bootstrap replications [23,24]. The percentage identities of nucleotide and amino acid sequences of ALSV were computed in MEGA 10/11 using the default settings.

### 2.5. Nucleotide Sequence Accession Numbers

Nucleotide sequences determined in the study are available in the GenBank database under accession numbers: PP623704–PP623718 and PP942935–PP942941 for Segment 1; PP623719–PP623733 and PP942942–PP942948 for Segment 2; PP623734–PP623748 and PP942949–PP942955 for Segment 3; PP623749–PP623763 and PP942956–PP942962 for Segment 4.

### 2.6. Biosafety

Experiments with potential infectious material were carried out in accordance with the requirements of the following biosafety rules: “Sanitary and Epidemiological Requirements for the Prevention of Infectious Diseases” N 3.3686–21 dated 28 January 2021.

## 3. Results

### 3.1. Tick Collection and ALSV Detection

The study analyzed 4458 individual samples of adult ticks collected from 22 regions of Russia (Figure 1, Tables S1 and S2). Among them, 22 ticks were found to be ALSV-positive by RT-PCR; the average infection rate of ticks was thus 0.5% (22/4458; 95% CI: 0.3–0.7). ALSV-positive ticks were detected in the Transbaikal Territory, Irkutsk Oblast, Tuva Republic, Republic of Khakassia, Kemerovo Oblast, Udmurt Republic, and Vologda Oblast (Table S2). The highest infection rates were in the Republic of Khakassia (3.3%) and Kemerovo Oblast (2.4%). The lowest infection rate was in Vologda Oblast (0.4%). In this study, all ALSV-positive ticks belonged to the species *I. persulcatus*. No positive samples were found among the *I. ricinus* ticks studied from the Bryansk and Smolensk oblasts, although ALSV-positive *I. ricinus* ticks have been detected in Russia [20].

### 3.2. Analysis of Genome Identity

When compared with other ALSV isolates found in China, the studied isolates from the Asian part of Russia have a nucleotide sequence identity level of about 96.5% for Segments 1, 3, and 4, and 94.5% for Segment 2. The corresponding figures for Russian isolates of the European clade are in the range of 90% for segments 1, 3, and 4, and 91% for Segment 2. The level of difference with the prototype isolate found in Finland in the *I. ricinus* tick is in the 89–93% range.

**Figure 2:**
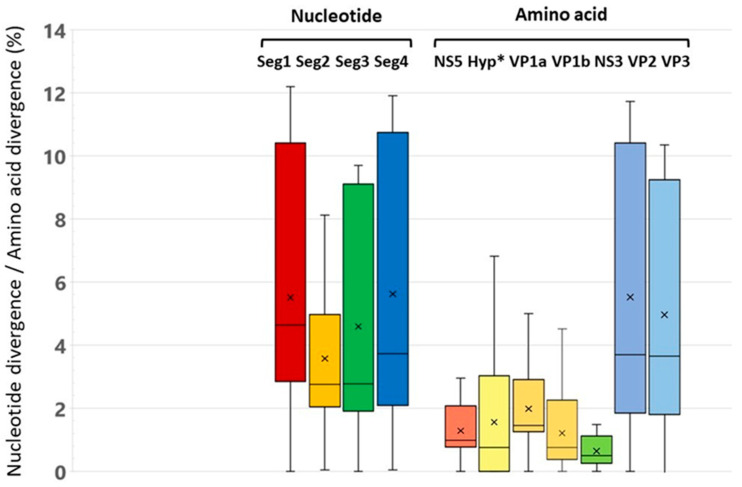
The level of difference in nucleotide and amino acid sequences for genomic segments and viral polypeptides of the studied ALSV isolates. * for the entire amino acid sequence of the Segment 2 (encoded VP1a and VP1b).

The level of nucleotide sequence differences between the studied genetic variants of ALSV is about 5% for Segments 1 and 4 and about 4% for Segments 2 and 3 (Figure 2). Differences in deduced amino acid sequences for proteins encoded by Segments 1, 2, and 3 (NS5, VP2, VP3, VP1a, VP1b, and NS3) were roughly 1%, with the highest variation observed in VP2 and VP3 encoded by Segment 4. The most conserved proteins were nonstructural proteins NS3 and NS5.

Heat maps of identity for nucleotide and amino acid sequences for the described and studied ALSV isolates also demonstrate the above-described difference between the compared sequences (Figures S1 and S2). However, it is noteworthy that nucleotide substitutions characteristic of all segments do not always lead to pronounced amino acid substitutions. Moreover, the amino acid sequences of NS3 and VP2 have the greatest conservatism, and the variability is typical for the VP1a polypeptide.

### 3.3. Phylogenetic Analysis

**Figure 3:**
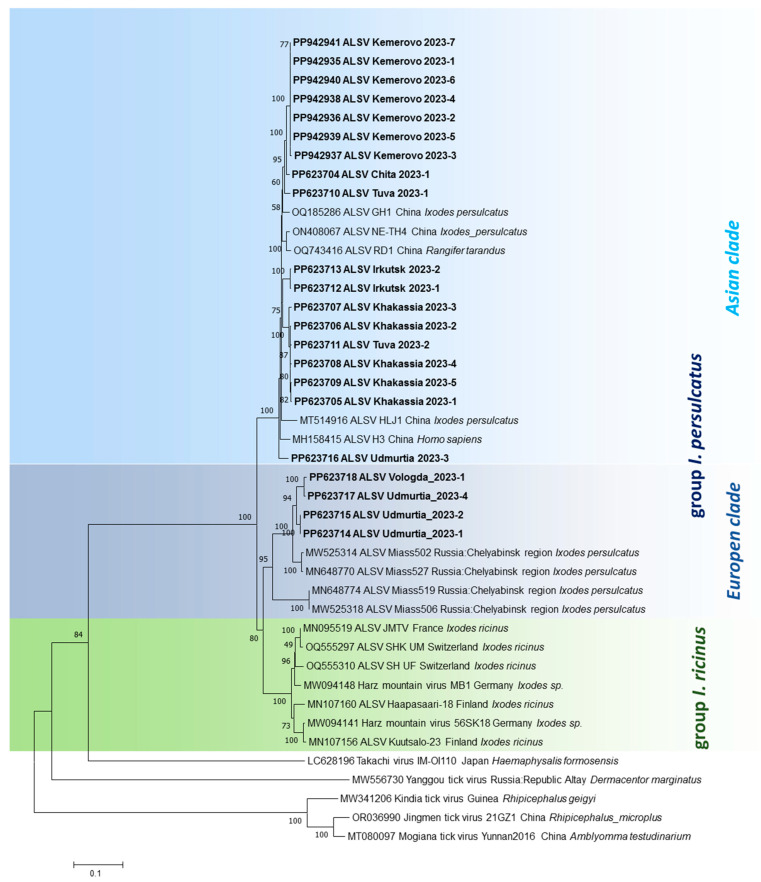
Phylogenetic tree of ALSV variants for Segment 1 (NS5-like gene). Sequences sequenced in this study are shown in bold. The phylogenetic trees of Segments 2–4 are similar.

The phylogenetic trees demonstrate that genetic variants of ALSV circulating in *I. persulcatus* ticks in the southern ranges of Eastern Siberia (the Transbaikal Territory, Irkutsk Oblast, Tuva Republic, and the Republic of Khakassia) and Western Siberia (Kemerovo Oblast) are grouped with sequences found in China across four segments (Figure 3). This Asian subtype (clade) is represented by variants that form the Asian isolates found in *I. persulcatus* ticks (and in humans). Interestingly, this clade also includes a single isolate from the Udmurt Republic (Europe), which is located on the border between the European and Asian parts of Russia.

Most of the ALSV variants from the Udmurt Republic, as well as the isolate from Vologda Oblast, belong to separate clades within the European subtype, together with prototype variants from Chelyabinsk Oblast (Ural Mountains). These isolates were detected in *I. persulcatus* ticks. Another clade of the European subtype is associated with *I. ricinus* ticks and is found in Western European regions.

## 4. Discussion

The current epidemiological situation in Russia with regard to tick-borne infections is characterized not only by multiple incidents of already known tick-borne infections, but also by the detection novel tick-borne pathogens such as ALSV. ALSV was first isolated from the blood of patients with fever in northeastern China [13,14]. The virus’ RNA was detected in 86 of 384 patients with fever and those with a history of tick bites. Patients infected with ALSV had a history of fever, headaches, and other symptoms that resemble the manifestations of other tick-borne infections. Closely related viruses such as Jingmen tick virus, Mogiana tick virus, and Kindia tick virus have also been detected in primates in Uganda, cattle in Brazil and Guinea, and patients with Crimean–Congo hemorrhagic fever in Kosovo and Russia [8,25,26,27,28,29].

The ALSV genome is represented by ssRNA of positive polarity and consists of four segments [13,14]. Recently, the two novel putative structural proteins in the duplicated segments have been described [12]. This result highlights the fluid nature of the genomes of Jingmenviruses and their multipartite virions. Different combinations of segments packaged in different virus particles could facilitate the acquisition or loss of genomic segments and segment duplication following genomic drift. Comparison of the nucleotide sequences revealed high intraspecific variability at a level of 4.6–7.7%. Amino acid sequences were more conserved, with 0.5–1.9% variability. The NS5 and NS3 flavi-like proteins, encoded by Segment 1 and 3, respectively, are the most conserved polypeptides. The exceptions are the structural glycoproteins VP1a and VP1b, in which the amino acid variability reaches 7.5% and 4.5%, respectively. The increased variability of the putative structural proteins may be due to the pressure of the host immune response or the need for ALSV to adapt to different hosts. The accumulation of point substitutions in these proteins probably provides ALSV with the ability to replicate in various hosts and in different natural foci. Analysis of complete nucleotide sequences of the four segments of the ALSV genome showed that the identity level of the nucleotide sequences (4–6%) of Asian isolates is closer to ALSV isolates previously found in China. The European ALSV isolates have a greater number of differences that indicate the independent evolution of ALSV in different geographical regions of Eurasia.

Phylogenetic analysis of the four genome segments of ALSV showed that ALSV isolates may be divided into Asian and European subtypes (Figure 3). The Asian subtype is closely related to isolates first isolated in China, close to the Russian–Chinese border [13,14], and these isolates are associated with the novel ALSV variants found in this study in the southern ranges of Eastern and Western Siberia. All of these isolates were found in *I. persulcatus* ticks only. The ALSV isolates of the European genotype are associated with two species of ticks, *I. persulcatus* and *I. ricinus* [19,20,21]. These isolates form two separate phylogenetic branches (subclades). They can provisionally be divided into Western European and Eastern European subtypes of ALSV, with the latter including isolates collected in the Ural Mountains. It may be conjectured that the ecosystems of the south of Eastern Siberia and the north of Mongolia are optimal for the circulation ALSV infection [13,30]. Moreover, the vast territory the southern reaches of Eastern Siberia border is territorially close to the interior regions of China, where the circulation of ALSV was firstly detected [14,31].

Previously, ALSV isolates were divided into the *I. ricinus* and *I. persulcatus* groups according to the main vector species. The *I. persulcatus* group is divided into two subgroups, the European (the republics of Karelia and Altai and Chelyabinsk Oblast) and the Asian (China, the republics of Altai, Tuva, and Karelia, the oblasts of Chelyabinsk and Ulyanovsk, and Altai Krai) [19,20,21]. This assumption was confirmed in the present study. All ALSV isolates circulating in the south of Eastern Siberia and in Western Siberia in *I. persulcatus* ticks were clearly clustered into the Asian subgroup of the corresponding vector when analyzed for each of the genome segments. Of interest is a territory in the Udmurt Republic, where most ALSV variants are clustered into the European branch, but an isolate attributed to the Asian branch is also encountered.

Russia tends to experience persistent foci of tick-borne infections in urban and suburban areas [5,29,32,33]. Ticks inhabiting city parks and squares are especially dangerous, since city dwellers perceive the urban environment as being free of ticks and do not take any non-specific preventive measures, unlike people visiting natural biotopes. In our work, a number of places where ticks with ALSV RNA was detected can be classified as biotopes with a high anthropogenic load and located within rural settlements (for example, in the Vologda Oblast, Udmurtia, and Kemerovo Oblast) or along busy highways, as in the Irkutsk Oblast. Some places where ticks with ALSV RNA were detected in Udmurtia are located near a children’s country camp.

## 5. Conclusions

The study shows the wide distribution of ALSV in Russia. The highest levels of virus detection were in the Asian part of Russia, and ALSV genetic markers were predominantly associated with *I. persulcatus* ticks. Analysis of the complete nucleotide sequences of the four segments of the viral genome showed that the nucleotide sequences of the Asian isolates exhibited a high identity to ALSV isolates previously found in China. The highest level of differences is observed for the VP2 and VP3 polypeptides (Segment 4). Flavi-like proteins NS5 and NS3, encoded by Segments 1 and 3, respectively, are the most conserved polypeptides. This information, together with phylogenetic analysis for the four genome segments of ALSV indicate the existence of Asian and European subtypes (clades) that may be associated with *I. persulcatus* and *I. ricinus* ticks, respectively.

These data make evident the need to detect changes at the boundaries of the spread of modern ALSV isolates and other flavi-like viruses that are potentially dangerous to humans, which will make it possible to predict the transmission of these tick-borne infections.

## Figures and Tables

**Table 1:** Panel of primers for targeted library enrichment sequences of different segments of the ALSV genome for NGS.

Description	Primers	Sequences (5′–3′)	Size (bp)
Segment 1	AL1_1F	GCCATGATTGTCCTGATAGTG	
	AL1_1R	GCCCTGTCCATCTTCATTTCC	982
	AL1_2F	AGGAAAGACAGATCACTCAC	
	AL1_2R	GGACATCATGGACTTCTCCT	1038
	AL1_3F	AGAAGTCCATGATGTCCTCC	
	AL1_3R	GTTCATCCAGTCCTTGTAGTTTCC	840
Segment 2	AL2_1F	GTAACCTCCGTAGACTGTCCA	
	AL2_1R	GTCCCTTCCGTTTGGTTGTG	477
	AL2_2F	CTTGCTACATCGGAATCATGCC	
	AL2_2R	GATAAGCCCTCTCGATACCTC	1091
	AL2_3F	TGGTACGACTGGCTTTCGAG	
	AL2_3R	ACTTGTTGTAGTCTGCAACCC	1098
Segment 3	AL3_1F	TCGTCCAAGACTACTTAACAG	
	AL3_1R	GTATCGCCTGTCCTCTATCC	721
	AL3_2F	TGCTGTCCATAGCAATCATACC	
	AL3_2R	GTAGGACACGTCCTTTGCGA	865
	AL3_3F	GCAAAGGACGTGTCCTACGT	
	AL3_3R	TTACCACTTGCTGGTCACAG	1314
Segment 4	AL4_1F	ACTTTGATCTACATCCTCGCC	
	AL4_1R	GTATCCAGCTCTTCCCTTCTC	824
	AL4_2F	GGAAGAGCTGGATACCGAACTG	
	AL4_2R	TGCCAGATGTGTAGCTTCCC	1274
	AL4_3F	CAGCACTGGCGAAGATAACC	
	AL4_3R	TGCCCTGATACCTCCTAGCA	503

Note: the primer hybridization temperature was 58 °C.

## Data Availability

The original contributions presented in this study are included in the article and Supplementary Material. The ALSV genomes are published in GenBank with the accession codes: PP623704–PP623763 and PP942935–PP942962. Further inquiries can be directed at the corresponding author.

## Supplementary Materials

The following supporting information can be downloaded at: https://www.mdpi.com/article/10.3390/microorganisms13112564/s1, Table S1: Characteristics of the studied tick samples and the number of PCR-detected ALSV-positive ticks; Table S2: List of the collection sites of ALSV-positive ticks detected by RT-PCR; Figure S1. Heat maps of identity (%) for nucleotide sequences of different ALSV isolates. (a) For Segment 1 encode NS5-like sequences; (b) for Segment 2 encode VP1a and VP1b sequences; (c) for Segment 3 encode NS3-like sequences; (d) for Segment 4 encode VP2 and VP3 sequences; Figure S2. Heat maps of identity (%) for amino acid sequences of different ALSV isolates. (a) For Segment 1 encode NS5-like sequences; (b) for Segment 2 encode VP1a and VP1b sequences; (c) for Segment 3 encode NS3-like sequences; and (d) for Segment 4 encode VP2 and VP3 sequences.

## Abbreviations

The following abbreviations are used in this manuscript:

ALSV	Alongshan virus
ssRNA	single-stranded RNA
dsDNA	double-stranded DNA
