# Supplementary material for: Diversity of the Alongshan Virus in Ixodes Ticks Collected in the Russian Federation in 2023
Source: Microorganisms. 2025 Nov 10;13(11):2564. doi: 10.3390/microorganisms13112564 (PMC12654158; doi:10.3390/microorganisms13112564)
Supplement: Supplementary file 1 [file microorganisms-13-02564-s001.zip › microorganisms-3900497-supplementary.pdf]

Table S1. Characteristics of the studied tick samples and the number of PCR–detected ALSV positive ticks.

Regions	Tick species	Total tested ticks	Positive samples, infection rate
Altai Republic	*I. persulcatus*	79	No ALSV positive
Arkhangelsk Oblast	*I. persulcatus*	428	No ALSV positive
Bryansk Oblast	*I. ricinus*	51	No ALSV positive
Irkutsk Oblast	*I. persulcatus*	200	2/200; 1,0%; 95% CI: 0,3–3,6
Ivanovo Oblast	*I. persulcatus*	135	No ALSV positive
Kemerovo Oblast	*I. persulcatus*	289	7/289; 2,4%; 95% CI: 1,2–4,9
Khabarovsk Krai	*I. persulcatus*	79	No ALSV positive
Khakassia Republic	*I. persulcatus*	150	5/150; 3,3%; 95% CI: 1,4–7,5
Kirov Oblast	*I. persulcatus*	85	No ALSV positive
Komi Republic	*I. persulcatus*	82	No ALSV positive
Krasnoyarsk Oblast	*I. persulcatus*	200	No ALSV positive
Mari El Republic	*I. persulcatus*	55	No ALSV positive
Novosibirsk Oblast	*I. persulcatus*	107	No ALSV positive
Primorsky Krai	*I. persulcatus*	368	No ALSV positive
Smolensk Oblast	*I. ricinus*	285	No ALSV positive
Tomsk Oblast	*I. persulcatus*	340	No ALSV positive
Transbaikal Territory	*I. persulcatus*	150	1/150; 0,7%; 95% CI: 0,1–3,6
Tver Oblast	*I. persulcatus*	170	No ALSV positive
Tyumen Oblast	*I. persulcatus*	137	No ALSV positive
Tuva Republic	*I. persulcatus*	210	2/210; 0,9%; 95% CI: 0,3–3,4
Udmurt Republic	*I. persulcatus*	600	4/600; 0,7%; 95% CI: 0,3–1,7
Vologda Oblast	*I. persulcatus*	258	1/258; 0,4%; 95% CI: 0,1–2,2

Table S2. List of the collection sites for ALSV positive ticks detected by RT PCR.

Isolate name	Site where ticks was collected	Geographic coordinates (N; W)
Chita–1	Duldurginsky District, Alkhanai National Park	50.9031551; 113.1867298
Khakassia–1	Republic of Khakassia, Beysky District, on the Tabat River	52.9314589; 90.7455396
Khakassia–2	Republic of Khakassia, Beysky District, on the Tabat River	52.9256640; 90.6624555
Khakassia–3	Republic of Khakassia, Beysky District, on the Tabat River	52.8991732; 90.7408499
Khakassia–4	Republic of Khakassia, Beysky District, on the Tabat River	52.8927855; 90.6816187
Khakassia–5	Republic of Khakassia, Sayanogorsk, dacha community Bolshoy Karak	53.0171084; 91.4599841
Tuva–1	Republic of Tuva, Todzhinsky district, Tozhu pass	52.4357081; 96.4813861
Tuva–2	Republic of Tuva, Todzhinsky district, Tozhu pass	52.4318358; 96.5114108
Irkutsk–1	Irkutsk Oblast, Baikal tract	52.0647073; 104.6066136
Irkutsk–2	Irkutsk Oblast, Baikal tract	52.0321271; 104.6454436
Kemerovo–1	Yashkinsky municipal district, village Pisanaya	55.308444; 86.159361
Kemerovo–2	Yashkinsky municipal district, village Pisanaya	55.308444; 86.159361
Kemerovo–3	Yashkinsky municipal district, village Pisanaya	55.308444; 86.159363
Kemerovo–4	Kemerovo municipal district, village Podyakovo	55.571806; 85.824028
Kemerovo–5	Kemerovo municipal district, village Podyakovo	55.571806; 85.824028
Kemerovo–6	Kemerovo municipal district, village Podyakovo	55.571806; 85.824029
Kemerovo–7	Kemerovo municipal district, village Podyakovo	55.571806; 85.824029
Udmurt–1	Republic of Udmurtia, Votkinsk district, children's camp «Chaika	57.102109; 53.941665
Udmurt–2	Republic of Udmurtia, Votkinsk district, children's camp «Chaika»	57.102109; 53.941665
Udmurt–3	Republic of Udmurtia, Votkinsk district, children's camp «Chaika»	57.102110; 53.941666
Udmurt–4	Republic of Udmurtia, Glazovsky district, village «Simashur»	58.193896; 52.739647
Vologda–1	Vologda Oblast, Velikoustyugsky district, village «Krasnoe pole»	60.783251; 46.237931

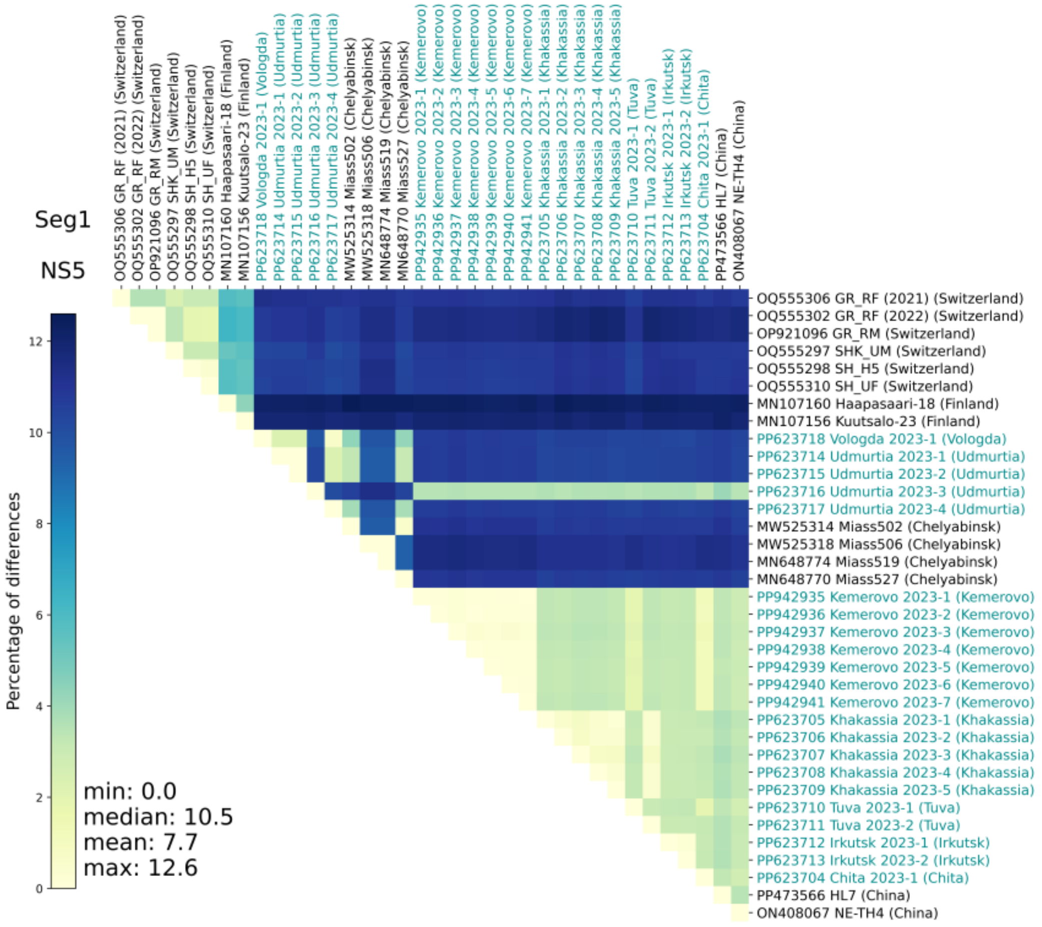

(a) Segment 1, NS5

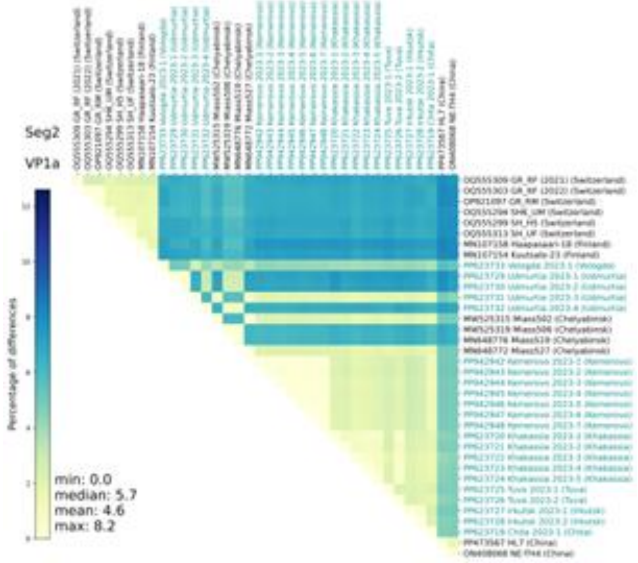

(b) Segment 2, VP1a

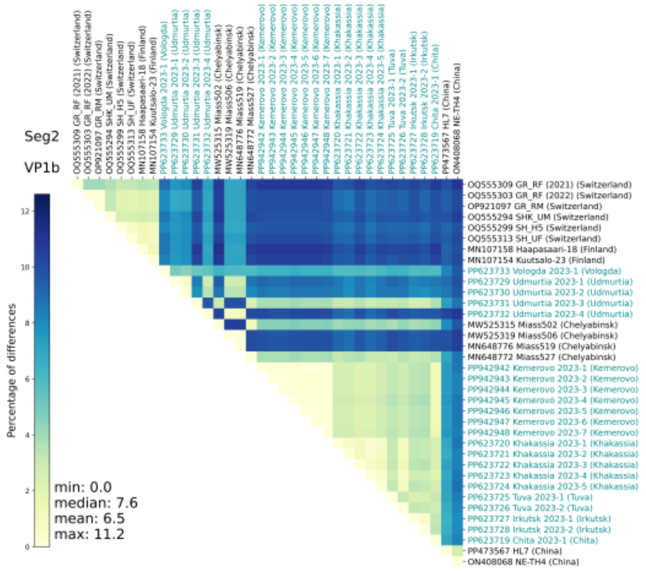

Segment 2, VP1b

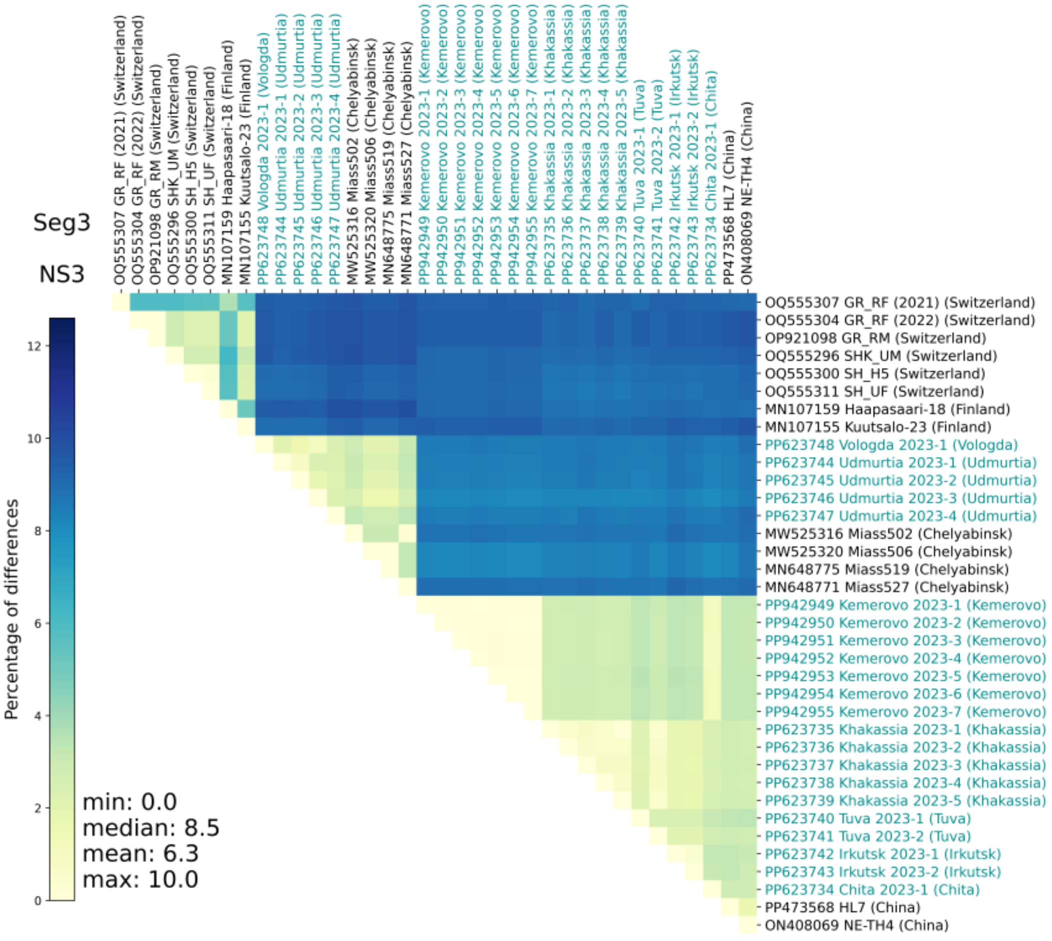

(c) Segment 3, NS3

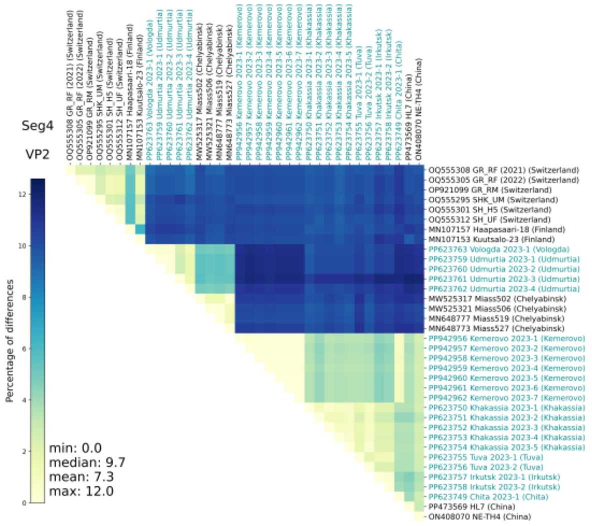

(d) Segment 4, VP2

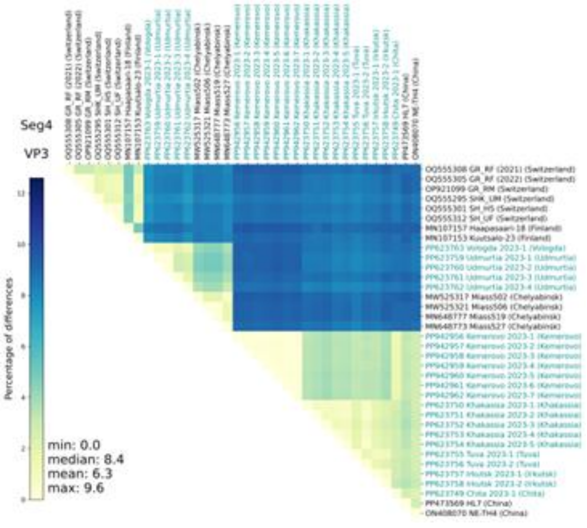

Segment 4, VP3

Figure S1. Heat maps of identity (%) for nucleotide sequences of different ALSV isolates. (**a**) For Segment 1 encode NS5-like sequences; (**b**) for Segment 2 encode VP1a and VP1b sequences; (**c**) for Segment 3 encode NS3-like sequences; (**d**) for Segment 4 encode VP2 and VP3 sequences.

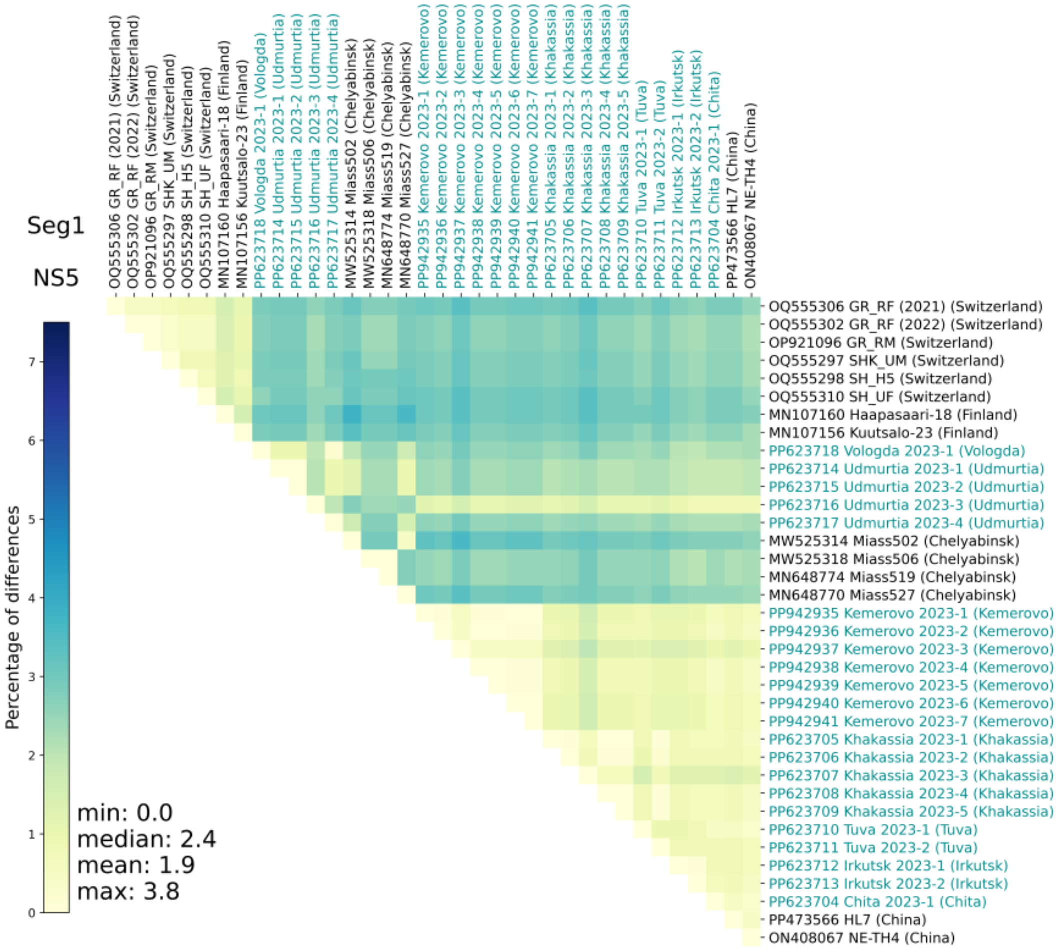

(a) Segment 1, NS5

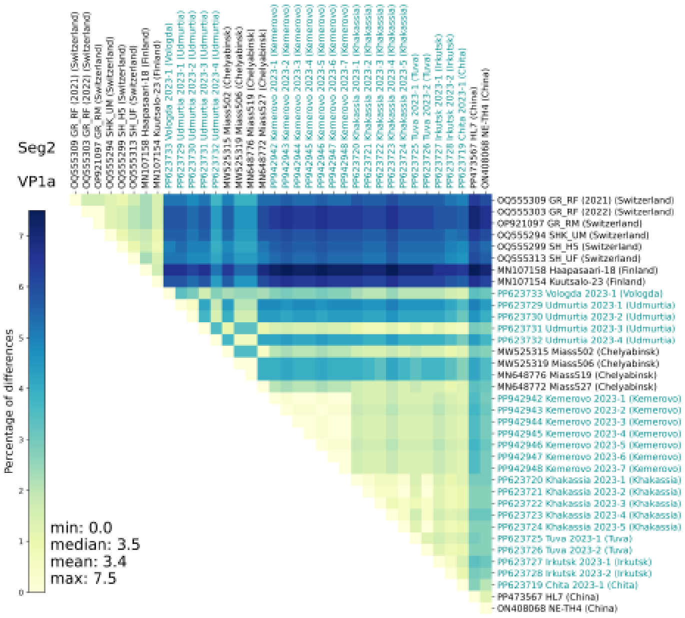

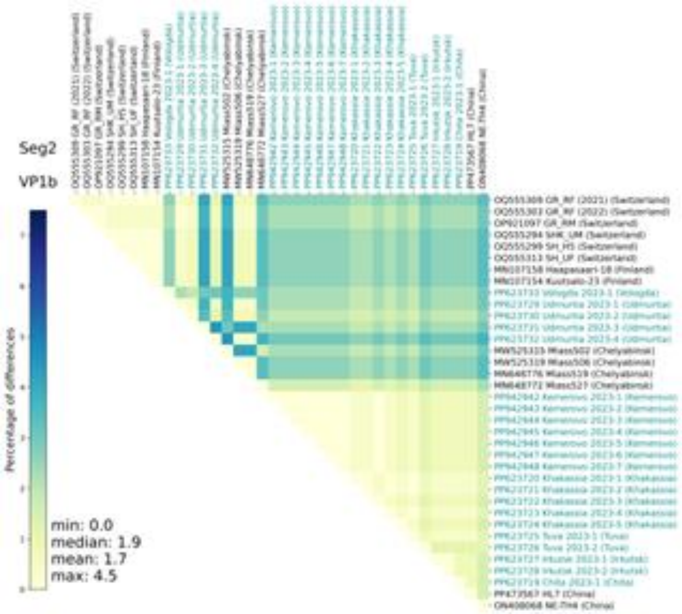

(b) Segment 2, VP1a **Segment 2, VP1b**

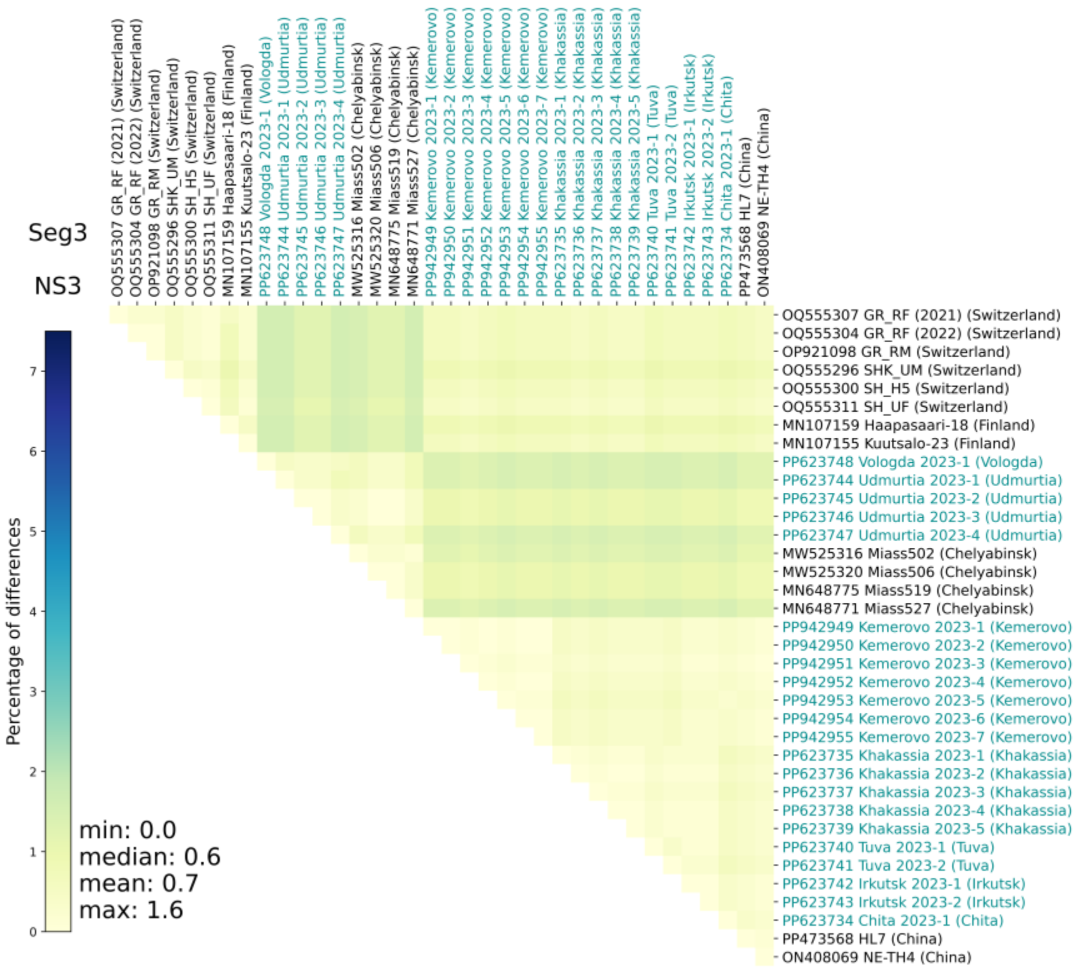

(c) Segment 3, NS3

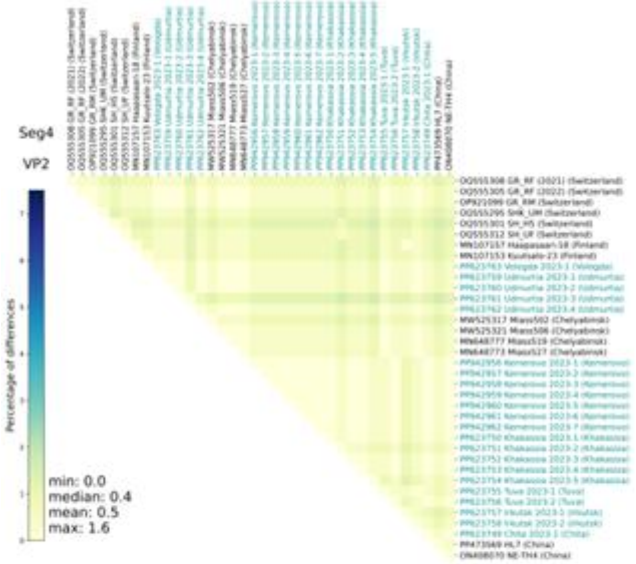

(d) Segment 4, VP2

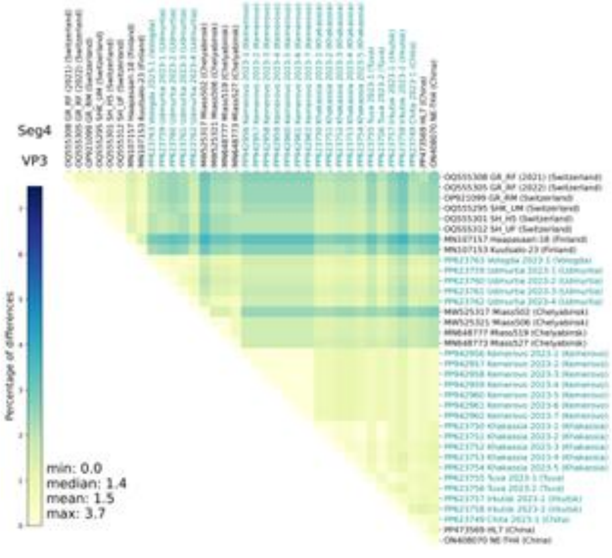

Segment 4, VP3

Figure S2. Heat maps of identity (%) for amino acid sequences of different ALSV isolates. (**a**) For Segment 1 encode NS5-like sequences; (**b**) for Segment 2 encode VP1a and VP1b sequences; (**c**) for Segment 3 encode NS3-like sequences; and (**d**) for Segment 4 encode VP2 and VP3 sequences.
